# How Society Changes: Sociological Enlightenment and a Theory of Social Evolution for Freedom

**DOI:** 10.1007/s12108-020-09464-y

**Published:** 2020-10-27

**Authors:** Mitsuhiro Tada

**Affiliations:** Faculty of Humanities and Social Sciences, Kumamoto University, 40-1, Kurokami 2 chome, Chuo-ku, Kumamoto, 860-8555 Japan

**Keywords:** Niklas Luhmann, Emile Durkheim, Alfred Schutz, Path-dependency, Eigen-time of self-referential system, Contingency

## Abstract

This article clarifies the relationship between individual freedom and social order by relying on Niklas Luhmann’s social systems theory and thereby defines sociology’s contribution to social evolution as *sociological enlightenment*, which seeks otherwiseness in living experience and action. For this purpose, Luhmann’s theory will specifically be compared with Emile Durkheim’s and Alfred Schutz’s sociological theories. Durkheim, a “child of the Enlightenment,” considered freedom a collective ideal of moral individualism and conceived that the rational state realizes freedom by spreading the civil-religious human ideal for modern social order. In contrast, Schutz, following Henri Bergson, who criticized rationality for spatially fixing inner time, regarded freedom as a given in the individual’s underlying duration, not as a shared ideal. Yet, unlike Bergson, he continued relying on rationalism, and he thought that the sociological observer observes how something appears to people with the epoché of natural attitude, not what it objectively is. Inheriting this phenomenological subjectivism, Luhmann showed that the self-referentiality of consciousness also applies to society: A social system, which path-dependently emerges itself from a double contingency, observes the world in its own way based on its self-referentially constituted eigen-time. On account of this system closure, and contrary to Durkheim’s illuminist belief, there is no controlling entity in a highly evolved society, where freedom results from the enlarged, diversified possibilities of living experience and action (contingency). Thus, sociological enlightenment doubts self-evidence so that society brackets the taken-for-granted social order or social reality and amplifies individuals’ deviations to evolve toward freedom.

*[T]he history of the human spirit is the very history of the progress of free thought.*

—Émile Durkheim (1897: 430).

*[A]ll free-living systems are nonequilibrium systems.*

—Stuart Kauffman (1995: 21).

## Introduction

This paper aims to clarify the relationship between individual freedom and social order, thereby redefining the role that sociology can play for further freedom in society. For this purpose, the following argument specifically relies on Niklas Luhmann’s theory of social systems, which characterized sociology’s contribution as *sociological enlightenment*, an enlightenment that enlarges the room for new kinds of “living experience (*Erleben*)” and “action (*Handeln*)” by revealing the path-dependency of the existing, taken-for-granted social order or social reality (cognitive order). Any social order or reality is merely selective and therefore could be otherwise.

Jürgen Habermas famously criticized Luhmann’s systems theory for explaining “only how society changes (*wie Gesellschaft sich ändert*), but not how society *should* change (*wie sie sich änderen soll*)” (Der Spiegel 1971: 205, emphasis added): systems theory does not provide norms for what one should do about the existing social system; it is therefore a social technology that contributes to the stabilization of dominance, abandoning the practice of a free consensus of citizens concerning social reforms (Der Spiegel 1971: 205). This condemnation recalls Karl Marx’s aphorism in his *Theses on Feuerbach*. There are surely cases in which changing society is more important than interpreting (understanding) it. However, the impossibility of centralist planning (teleology) of a complex society has become apparent through the practice of Marxism. Even if society could be successfully changed based on such rationalist planning, the changed society would neither always be ideal for everyone nor remain in that condition without other changes in the future. In any case, the belief that there is a single normatively correct order, whether illuminist or obscurantist, could severely restrict freedom. Today, especially, since both the left and the right are keen to change society in some form, it seems meaningful to theoretically clarify how society changes on earth, apart from the encouragement of practice based on a particular normative ideal. Considering this issue in relation to individual freedom is the main aim of this article.

For this purpose, I focus on Luhmann’s conception of social evolution. For Luhmann, the lack of a privileged entity controlling all of society was evidence of a highly evolved society that has great complexity and also *a considerable degree of contingency of the individual’s living experience and action*; individual freedom is socio-evolutionarily achieved as enlarged otherwiseness in living experience and action. His sociological enlightenment referred to doubting realities that are taken for granted in modern life so that society can bracket the path-dependent existing order and amplify deviations derived from the fluctuations of individuals. Sociology’s task is not only to elucidate how social order gets constituted. It is also to indicate that the existing order is merely an accidental result of path-dependency and is therefore neither absolute nor invariant. In paying attention to this contingency of social order, Luhmann seems to have thought that sociology could help society to change itself and evolve toward freedom by showing other possible ways of life, not by instructively guiding to the sole correct(−seeming) answer for social change.

In the following discussion, I demonstrate this interpretation through a comparison to the ideas of other sociological theorists, specifically Emile Durkheim and Alfred Schutz, by occasionally applying system-theoretic terms to their thoughts for explanation and by referring to Henri Bergson’s philosophy as a bridge. As detailed later, Durkheim’s and Schutz’s theoretical considerations focus on the relationship between freedom and order in modernity and provide meaningful considerations toward the aim of this article.

## From Normative Order to Spontaneous Order

### The Soul of Society: Durkheim’s Theory of Normative Order

Luhmann’s theory of social systems is often critically characterized as post-modernist, counter-Enlightenment (see, for instance, Habermas 1971: 161–162). In reality, he had rather conceived himself to inherit the task of the Enlightenment since the beginning by proposing sociological enlightenment that reflexively clarifies the limits of the old-fashioned, reasonable Enlightenment (Luhmann [1967] 1970: 67–68). Nevertheless, the notion of sociological enlightenment that he proposed seemed to have remained unclear. Luhmann only formulated the core meaning of sociological enlightenment abstractly as “the increase in human potentials for the grasp and reduction of world complexity through system-building” (Luhmann [1967] 1970: 78), adding “Unlike the reasonable Enlightenment, sociological enlightenment will no longer search for fixed, intersubjectively certain truths of reason and deduce everything else from them” (Luhmann [1967] 1970: 79). These statements might lead to misunderstanding of sociological enlightenment as a part of social technology of state technocrats for stabilizing the existing system (or the *status quo*) (see, for instance, Habermas 1971: 260). The following discussion will show the opposite implications of sociological enlightenment that will contribute to the evolutionary change of society for individual freedom. Therefore, I first cite as a comparative example a classic who attempted to legitimate the Enlightenment by sociology: Emile Durkheim, a “child of the Enlightenment” (Hughes 1958: 280), who lived in the French Third Republic.

Through his analysis on *ideals*, Durkheim presented a unique sociological insight into the co-evolutionary relationship between individual freedom and social order in modernity. In his opinion, sociology itself never constructs an ideal; the ideal is a social reality given as the collective consciousness that is already at hand, and as such is a research object of sociology (Durkheim [1911] 1951: 136, 140–141). In this French sociologist’s mind, individualism had become a *civil religion*: individualization in modernity had made the person into the sacred thing, which works as a bond of organic solidarity in a diversified society (Filloux 1994: 19–20). Durkheim states,

[O]ne of the fundamental axioms of our morality—*the* fundamental axiom, one could say—is that the human person is the sacred thing par excellence. […] According to this principle, any kind of encroachment on our heart of hearts appears to us as immoral since it is a violence against our personal autonomy. (Durkheim [1925] 2012: 112, emphasis added)

He thought that *the evolution of the ideal* into the worship of human beings, which he calls *moral individualism*, is a necessity correlated with a change in social structure, stating that “All moral systems actually practiced by the people are a function of the social organization of these peoples, and stem from their [the people’s social] structure and vary with it” (Durkheim [1906] 1951: 81). Human-historically viewed, the evolution of ideals from naturalistic or animistic beliefs to civil-religious universal ideas such as freedom and equality is linked with the structural change from a primitive to a modern society (Durkheim 1912). According to Durkheim, individualization and diversification coupled with the division of labor in industrializing society lead to an idealization of abstract humanity, that is, of “the dignity of mankind” (Durkheim [1925] 2012: 33) as inviolable; as a result, individual freedom and social order become compatible.

Both social division of labor and the evolution of the ideal into this “cult of the human person” are part of *social evolution* (see Filloux 1994: 19–20). This aspect of Durkheim’s thought differed, on the one hand, from that of optimistic liberalists such as Herbert Spencer, who believed that society, as an organism, evolves through natural selection and the survival of the fittest. Such egoistic individualism approving the struggle for existence (“the war of all against all”) would create neither social capital (trust) nor “fellow feeling” and thereby disturb the development of the social division of labor. In contrast, moral individualism functions as the “non-contractual element in contract” (Talcott Parsons), based on which diversified people exchange different goods and services with each other in industrial society. Durkheim says that if we imagine

[…] that it will be enough […] to transport into sociology the better-known laws of biology by plagiarizing them, we pay with deluding ourselves. If sociology exists, it has its own method and its own laws. (Durkheim [1888] 1987: 92).

To begin with, natural selection does not clarify how the diversity of specialities and individualities required for the division of labor can be achieved. As Hermann Haken, a physicist who is known for his theory of cooperative phenomena called synergetics, points out, the fact in the complex world of nature is that *symbiotic relationships* also exist: Bees mediate pollen in exchange for honey; flowers would become extinct if bees were wiped out (Haken [1981] 1990: chap. 8). Likewise, society is an organic system that becomes internally more interdependent as it becomes more complicated and professionally specialized; in consequence, people regarded as “weak” are also granted some role in contributing to society (see Durkheim [1893] 1973: 253–254). If this is the case, social Darwinism—neo-liberalism in today’s context—which wields the proverb “the weakest go to the wall,” would result in all going down in defeat together, with the nemesis reflexively bouncing around from one place to the next: society would devolve rather than evolve.

On the other hand, Durkheim differed from Marxists. In dealing with the issue of the fierce struggle for survival under capitalism, he did not appeal for a reform in the base of possessive relations. What seemed significant to him was that the ideal, the superstructure, evolves to moral individualism and people share it; this could not only prevent the anomalous division of labor fraught with serious labor-management confrontations, but also socialist despotism suppressing individual freedom. Under moral individualism, moreover, individual merits that one achieves on one’s own would remain respected, while social inequalities would also be eradicated (see Filloux 1994: 21–22). In short, the confrontation between freedom (meritocracy) and equality (socialism) will be bridged by humanistic fraternity (see Durkheim [1886] 1987: 203–204). Society thereby attains further evolution, maintaining order with solidarity: freedom and equality grow, along with industry.

Durkheim thus believed that respect for humanity in general is the ideal best suited for individualizing and diversifying modern society. For integration, society requires the sharing of an abstract ideal commensurate with its complex structure. Just as what makes a biological organism human is not the internal organs but the mind, the common ideal enables a social organism to establish a cooperative relationship among its functionally differentiated organs and to maintain the whole as a living society *sui generis*; Durkheim says, “Isn’t this ideal type, which each society asks its members to fulfil, the keystone of the whole social system and that which produces its unity?” (Durkheim [1906] 1951: 81–82). For this reason, Durkheim described the set of ideals animating society and enabling its internal solidarity as “*society’s soul* (*l’âme d’une société*)” (Durkheim [1925] 2012: 124, emphasis added). Note that this is different not only from religious morality before secularization (*laïcité*) but also from the people’s spirit (*Volksgeist*; *l’âme des peoples*) that was assumed in the German organicist notion of community (*Gemeinschaft*). Contrary to Ferdinand Tönnies’s assumption about morality in his *Gemeinschaft und Gesellschaft* ([1887] 1922), Durkheim believed that the moral order of *Gesellschaft* is superior to that of *Gemeinschaft*: The society’s soul—moral individualism—integrates civil society (*Gesellschaft*) as an organic whole despite its diversification, maximally realizing individuals’ freedom and equality (see Tada 2020).

This shows Durkheim to be a committed individualist and liberalist. He is often characterized as a representative sociologist of methodological collectivism or social realism, but he chose such a macro-theoretical stance so that he could advocate individual freedom in society. According to him, society realizes the ideal of freedom without leaving it at the level of speculation: “The theorist is allowed to demonstrate that a human being has the right to freedom (*liberté*); but, whatever the value of these demonstrations, it is certain that this freedom became a reality only in and through society” (Durkheim [1906] 1951: 79). Thus, he did not view society as always oppressive to individuals; in fact, it was the opposite.

Note, however, that Durkheim was primarily pointing to the integration of *French national society* through moral individualism; he stated, “Not only is individualism not anarchy; but it is henceforth the only system of beliefs that can ensure the moral unity of the country [France],” and “if there is a country among all others where the cause of [moral] individualism is truly national, it is our own” (Durkheim [1898] 1987: 270, 274). Thus, he even openly displayed his faith in the state (*l’État*) as the control entity of the social organism, regarding the modern state as *the organ of rationality* that was evolutionarily born to concentrate and unify social functions arising from the social division of labor (see Badie and Birnbaum [1979] 1982: 27–37). The civilized state is *the practical backing* for the ideal of moral individualism, civil religion. Contrary to the Spencerian theory of the night-watchman state that longs for a reduction in the role of the state, Durkheim conceived of the state (government) as “the social organism’s cerebrospinal system” (Durkheim [1893] 1973: 198), which rationally controls the social-organic whole for individual freedom.

Durkheim was a “high priest of the civil religion of the French Third Republic” (Bellah 1973: x) who tried to sociologically legitimate the illuminative, universal human ideals. His reaction to the Dreyfus Affair—a hate crime toward the minority in today’s context—also derived from civil loyalty to the national credo of modern France, not from an ethnic solidarity with Jewish brothers. Durkheim hoped that society would be changed by state regulations to realize the modern ideal:

It [moral individualism] is expressed theoretically in the Declaration of Human Rights […]; it is, however, far from being deeply rooted in the country [France]. […] [F]or instituting moral individualism, it is not enough to declare it and to translate it into beautiful systems [of thought]; society must be arranged [practically through the state] in such a way as to make this constitution possible and durable. […] [I]t is the state which creates them [individual rights], organizes them, and makes them into reality. (Durkheim [1950] 1995: 95)

For instance, Durkheim asserted that the state liberates the individual from a pre-modern collectivist life (Durkheim [1950] 1995: 93–94, 97–99); above all, the education system ought to be put under state supervision. School education must be conducted in conformity with the ideals of modern France (Durkheim 1922: 59–62). Thus, public schools controlled by the state are required, as a secular institution of socialization differing from family or church so that children, the next generation of French society, can cultivate their own individuality and also grow to respect others’ human dignity. Durkheim additionally conceived that international concord and the peace of mankind would be realized naturally, without the banner of cosmopolitanism, if an appropriate education of the universal ideal were provided in each country’s national framework (Durkheim [1925] 2012: 87–90).^1^

This aspect of Durkheim’s thought is a case of methodological nationalism, and politically that of *liberal nationalism*: like Will Kymlicka (2001), Durkheim had faith in modern Western values and ideals, the evolution of society toward their realization, and the need for state intervention in this process (see Adachi 2013: 67–90). In other words, his social theory was oriented toward the idealized order that he saw as normatively desirable.

However, on the other hand, note that Durkheim was aware of *the contingency (otherwiseness) of the ideal in society*. His assertion that the ideal is a given for sociological research implies that it is not an eternally unchanging constant in another world but a socially selected variable in this mundane world. Ideals are inherently *social* facts, no matter their contents. That being so, social theory must inquire more fundamentally into the social order underlying the choice of an ideal, as well as into the relation of this primary order with human individuals.

### Duration and Rationality: Schutz’s Theory of Spontaneous Order

For a more fundamental consideration of individual freedom and social order, I will next turn to Alfred Schutz. While Durkheim macro-sociologically conceived that freedom and order are highly compatible if people collectively share the ideal of moral individualism through socialization, Schutz found the possibility of freedom and order in the stream of sensory representations prior to conceptual thoughts including ideals, that is, in the *pure duration of consciousness*. Instead of sociality, he began with individual autonomy. However, before discussing Schutz, I will briefly refer to a French philosopher as a bridge for further discussion.

The person who especially provided Schutz with a theory of the primordial individual freedom based on conscious time was Henri Bergson. According to this contemporary of Durkheim, freedom (free will) exists in every individual’s fluid inner duration, not a fixed and shared ideal. Bergson famously dismissed the confrontation between determinism and indeterminism as an apparent problem resulting from the spatialization of time (Bergson [1889] 1908: chap. 3). When arguing free will, both determinists and their opponents spatially represent a stream that has already flowed past and then deny or defend the contingency of human action. After thinking back to the action’s starting point and reflectively visualizing other possibilities branching out from it, determinists allege that the action was predestined to be chosen because it was, in fact, selected over the others, while indeterminists conversely assert that the other actions were also possible because the action actually taken was still a considered choice. However, the time represented by both sides is always the one that has fictively flowed past, and is just an irreal, space-conceptually constructed symbol for the genuine lived time. That time is as good as a qualitatively homogenous time-scale in which neither actual human beings are living nor something creatively deviant from intelligence emerges. However far you follow the scale, you will not meet anything new there because it itself is merely a scale for calculation.

In reality, each of us is living in the continuous becoming of duration, which contains heterogeneous qualities that are irreducible to the quantitative measure; real human actions are also being taken in the currently flowing time prior to conceptual thinking. According to Bergson, this pre-conceptual pure duration is the genuine reality that is not yet subject to spatial fixing by intelligence; as such, it is the source of the evolution, which is unpredictably driven by impulse (*élan*). Evolution phenomena through something like revolution or innovation are neither explainable by teleology according to which the destination is pre-set in advance, nor by a mechanism according to which the subsequent state of the world is always determined by the preceding one. In each person’s pure duration, there are variant and fluid possibilities (otherwisenesses) as yet unfixed by concepts (ideals). This is what Bergson meant by freedom.

The free act takes place in time which is flowing, not in [conceptually fixed, irreal] time which has already flown. Freedom [*liberté*] is therefore a [given] fact, and, among the ascertained facts, there is none clearer. (Bergson [1889] 1908: 169)

Freedom is originally given at the sensory level of each individual. Contrary to Durkheim, Bergson had in this respect an element of *irrationalism, if not anti-intellectualism*, in his free thought: Bergson believed that modern intellectuality or rationality, although seen as the transcendent emancipator, paradoxically restrains individuals’ true freedom by reducing the original complexity of her/his inner time through its spatial simplification. Typically, positivism does this by intelligence; without seeing the genuine time of inner duration, modern science takes time as an independent variable and only repeats industrial manufacturing of the already-known, same object at best (Bergson [1907] 1940: 178–179, 363–396). That is, time descends into a mere calculating scale with which modern industry keeps efficient mass production. Bergson says, “Precisely because it [intelligence] always seeks to reconstitute, and to reconstitute with the given, intelligence lets get away what is *new* at each moment of history. It does not admit the unpredictable. It rejects all creation” (Bergson [1907] 1940: 177, emphasis in original). This was a reason why Bergson criticized modern-scientific rationalism for its reconstruction of time as the spatially calculable and insisted on a return to inner duration, which was given immediately to consciousness and not conceptually but only intuitively graspable. He also rejected Spencer’s evolution theory as a spatial reconstruction and treated *creative evolution*, an ongoing process that leaps ahead based on the *élan vital* in the present (see also Bergson [1907] 1909: 392–399).

Relying on Bergson’s insight, Alfred Schutz, the pioneer of phenomenological sociology, stated, “the problem of freedom, correctly understood, is purely a time problem” (Schütz 1932: 252). In system-theoretic terminology, the important thing here is, however, that inner time is *far from an “anything goes” random walk*. While the beginning is arbitrary, initial choices become self-bound and define the following process, to some degree. For instance, living experiences work themselves into the scheme of experience to determine the meaning of an ensuing living experience (Schütz 1932: 86–89). That is, to use a system-theoretic term again, *path-dependency* occurs in the course of consciousness (Tada 2013a: 254–256; Tada [2018] 2019).^2^ Then, Schutz himself, while approving this underlying, path-dependent duration, maintained Weberian action theory’s view of the modern individual who rationally acts toward the achievement of future goals s/he projects. An individual actor, as long as s/he is immersed in pure duration, is in the so-called “epoché of the natural attitude” (Schutz [1945] 1982: 229, 233) and has still not “awakened.” To be rational and genuinely autonomous, the actor must move away from inertial path-dependence and reflexively *plan* definite goals by intelligence and intermediate objectives by spatializing inner time (Tada 2013a: 118–119). As Schutz says, the unit of an ongoing action (*Handeln*) is “*a function of the span of the project* (*Entwurf*)” (Schütz 1932: 62, emphasis in original). Therefore, a sociology of modern society should explore unit acts uniquely defined by modern individuals themselves rather than diversified duration. The action’s aim that is rationally projected as a unit act is the action’s subjective meaning, which sociology deals with.

As shown here, the actual process of consciousness consists of both the diverse-remaining, non-reflexive duration and the uniquely defining, reflexive operation. Thus, Schutz thought that an actor successively experiences freedom and restraint in time consciousness.

A human being who lives and acts in the social world is a free being and performs her/his [projected] acts out of spontaneous activity. If such a [performed] action has passed, has completely come to an end, has fully become, and thus the act is resting in the finished and completed state, it is then, however, not free any longer but uniquely [*eindeutig*] determined. (Schütz 1932: 259–260)

Max Weber also suggested that the more freely one resolves to act, the more rational and therefore more nomological (or more deterministic) the action becomes (see Weber [1903–6] 1988: 132–137): when rational calculability exists, the projected goal hardly allows other actions to be chosen as means to the end. To pass an exam, you have no choice but to study.

However, even if a person’s immersion in pure duration, as Bergson believed, brings her/his conscious life to some sort of primordial freedom, it does not always lead to other people’s freedom in social life. Although the world is originally so complex that nobody can grasp the whole picture, an observer of the natural attitude automatically takes her/his view on others for granted as though it is the ontological reality. That is, s/he does not realize that s/he simplistically perceives others with *typifications* (or stereotypes). The same attitude would be often found among positivist scientists as well. Despite their selective perspective on the world, they themselves are inclined to believe that the world objectively exists as they observe it.

Such a naïve realism, even if unintentionally, often has a hand in discriminating against individuals who belong to vulnerable groups such as racial and ethnic minorities, women, sexual and gender minorities, and low-income populations (see also Schutz [1957] 1976): Naïve-realist majority (“we”) can strengthen biased views on such people in a weak position (“they”), sometimes by turning on the scientific-seeming grounds, and can even try to exclude strange elements from “our” social world by assuming that it is (and should be) homogeneous. However, as Schutz points out, “*Only the typical is homogeneous, but this always*” (Schütz 1932: 209, emphasis in original). Homogeneity assumed for drawing a boundary of “we” against “they” (or of “they” against “we”) is always an imaginary fiction. The real lifeworld which people inhabit is more diversified and not essentially dichotomized. While the “man on the corner” (and sometimes the “expert” as well) takes their heterogeneity-reduced, typified recognitions as the ontological reality, a person whom Schutz ([1946] 1976) calls the “well-informed citizen” attempts to reflect on her/his recognitions so that s/he knows the complexity of the social world. Rationality would imply this self-reflexive attitude out of path-dependent, pure duration: the attitude for civic-social life with others.

Note, however, that such a self-reflection also remains a *now and so,* which is selected autonomously in inner time and becomes itself a part of the duration again by sedimenting into the depths of consciousness. Schutz considered that such self-referentiality is of the essence of the conscious order. As for that “well-informed citizen,” too, Schutz carefully added that it precisely should denote “the citizen *who aims at being well informed*” ([1946] 1976: 122, emphasis added). The well-informed citizen is not an atemporal highest existence like god. S/he also lives in the mundane world, reproducing her/his own inner time, to which her/his reflections self-referentially belong. In this sense, the well-informed citizen is a self-enlightening process.

Schutz never fictionally postulates a superior (irreal), regulatory entity such as a transcendental subject. Without something transcendental, consciousness can self-referentially constitute its own order. Moreover, Schutz found that the same was true of the social order: even if individuals do not share some regulative ideal or value, the situation in the social world never falls into chaos.^3^ Citing Bergson, Schutz states: “Absence of order in the sense of *any kind of order at all* is, therefore, says Bergson, a meaningless expression, and refers only to the fact that an expected *particular* kind of order is wanting” (Schutz [1955] 1982: 300–301, emphasis in original; see also Bergson [1907] 1909: 238–258, 1934: 124–126). That is, the inversion of order as disorder is impossible. The counterpart of “order expected as desirable” is not disorder but “*spontaneous order*” (Schutz [1955] 1982: 301, emphasis added). “[A]nother kind of order [spontaneous order], irreducible to the former [an expected particular kind of order], prevails” (Schutz [1955] 1982: 301).

This view of Schutz’s on spontaneous order is much more comprehensive in sociological terms than Durkheim’s (and Talcott Parsons’s) normativist view of moral order. In fact, even when people of clashing ideals or values meet together, some order with a predictable course, to some extent, originates necessarily: war is a firm spontaneous social order that, once started, continues with high probability and, although undesired, is difficult to end. Thus, as Schutz does, we should ask with Bergson, “[W]hy there is order, and not disorder, in things” (Bergson [1907] 1909: 297): the kind of expected order that will be formed is indeed contingent, but the reality that order will be formed is necessary in and of itself.

## Contingency and Social Evolution

### Self-Referentiality and Fluctuation: Luhmann’s Contingency-Based Free Thought

Considering the discussion thus far, I shall enter into Luhmann’s systems theory.

Like Bergson and Schutz, Luhmann also thought of a system as autonomous and perceived that its autonomy was underpinned by temporal, path-dependent self-referentiality. For instance, according to Luhmann, a successive process consisting of system elements “generates through the intensification of selection, that is, *through the temporal restriction of the freedom degree of elements*” (Luhmann 1984: 610, emphasis added). A system element neither exists in itself alone like an atom nor appears at random, nor is it given by something external. Its meaning lies in relation to the other elements of the same system. That is, the meaning of an element is selectively determined as a *now and so* in the successive process (inner duration) consisting of other interrelated elements, and in turn, as a part of the process, it contributes to constitute new elements, giving a certain direction to the duration. Through such a self-regulative circulation, a self-referential system reproduces itself as *a becoming reality*, so to speak. Then, Luhmann, unlike Bergson and Schutz who focused on consciousness (conscious system), characteristically carried out theory-building at the macro-social level, defining the element of society as communication. Society, in his conception, is a spontaneous communicative order, dynamically stabilized on the basis of its own inner duration or *eigen-time*.^4^

In a world of chaos, due to the lack of weighting among occurrence probabilities, 100 % is evenly divided among infinite possible events; hence, they are all equally and extremely *improbable* (*unwahrscheinlich*) and it is, of course, impossible to pre-calculate which of them will be chosen. Everything would appear at random without interrelatedness. In contrast, in real society, some specific events become self-evident and *probable* (*wahrscheinlich*) through the self-reinforcing temporal development of a path, despite the contingency of its beginning. That is to say: *A path-dependent significant bias* (*a symmetry-broken iterative regularity*) that emerges *spontaneously* from the world of chaos (the symmetry of all event probabilities) is an order and, accordingly, a system (Tada 2013a: 253–257, [2018] 2019).^5^ It is precisely the disposition (trend) of choice self-formed through its own temporality.

Likewise, something transcendent such as an ideal or norm is also a function of the eigen-time of a real society and is accordingly a product of accidental path-dependence, that is, a selective thing (Tada [2018] 2019: 1006). This makes social criticism striving for alternatives possible but never means that society is in a defective condition with the ideal unfinished. In the case of a becoming reality, as Bergson ([1932] 1937: 260–261) indicated, it is a fallacy to believe the snapshot of an accidental resting state as essential and to look down on variability as defective. Furthermore, contrary to Durkheim’s illuminist assumption about the state initiative for the organic whole in the social division of labor, Luhmann thought that the characteristic of modern society is *the impossibility of a privileged authority controlling the functionally differentiated whole*: the absence of a ruling head (a cerebrospinal system) is evidence of a highly evolved society. The freedom and equality of all systems are achieved in the sense that neither ideal, norm, nor rational government can control the whole of society. More fundamentally, they are already found in the notion of self-reference: freedom is a given in the inner duration of each system in the form of closed, autonomous self-reproduction. Thus, for instance, deviation from social morality or rebellion against the government is theoretically possible in all circumstances.

However, Luhmann never ignored the reality that an individual’s choice is also conditioned by social structure. Therefore, how freedom becomes possible in real society must be discussed within the theoretical framework of a self-referential system; it is here that the social evolution enters the picture. Luhmann formulated *the increasing possibilities for living experience and action* (i.e., the growth of the number and variety of possible living experiences and actions) as the general tendency of social evolution (Luhmann 1971: 22). This expresses the development from a simple society to a complex society, and, in addition, freedom would be established when an actor can act differently based on her/his own choices, although evolution specifies behavioral regulations and the actor also knows them, which are path-dependently normalized (see Luhmann [1978] 2008: 120). In other words, freedom exists in a society in which complexity at the social level can be translated into contingency at the individual level, that is, into the selectability of an unconventional living experience or action. In this sense, freedom (*Freiheit*) “appears as the result of socio-cultural evolution” (Luhmann [1978] 2008: 120).

But a complex society, such as a functionally differentiated society, is not necessarily better than a simple one in terms of the degree of enlightenment and morality. For instance, given the exclusion from a particular functional system such as education, one could also be excluded from other functional systems and, because of anxiety and fear concerning such a life course, one might even democratically choose an authoritarian regime as a desirable order. Considering the rise of totalitarianism in modernity or the current situation of the world, the form of functional differentiation does not itself prevent society from turning toward a reduction of otherwiseness in living experience and action. Put differently, an increase in complexity does not always lead to an increase in rationality in this world (Luhmann 1975c: 214). Evolving into a better ideal is not promised. In terms of probability, the *devolution of society* is possible at any given moment. Even events that seem improbable, unless the probability is zero, can occur (and that sometimes democratically).

However, self-referential systems theory maintains the premise that an individual is operatively separated from other individuals and from society. There is no a priori connection supposed by concepts like intersubjectivity or culture; even socialization is self-socialization by the individual in question (Luhmann 1984: 327, [1985] 1995: 87, 2002: 52). Put simply, others are inherently black boxes, and there is no collective foundation leading to a perfect agreement or consensus. Only double contingency is always a given in social scenes. Since individuals exist autonomously based on their own inner durations, their living experiences and actions cannot be fully controlled from the outside. Yet, because of the necessary structural coupling with society, consciousness occupies a privileged position for intervention in communication (see Luhmann 1997: 114–115). Luhmann thought that these conditions allowed a spontaneous social order to emerge, as well as the eventual evolution of society: individuals are not agents that change society but rather a noise source outside society and, because of autonomy, *create fluctuations without necessarily intending to*. A fluctuation is a sort of deviant difference—a deconstructive shift, one might say—that brings about variants in society. Individuals can make society unstable and lively in this manner (see Luhmann 1978 [2008]: 83–84). For example, they can say “no” as well as “yes” in communication (see Luhmann 1997: 229–230, 2005: 202–204, [1990] 2008: 268–269). *Contingency-based heterogenization, rather than the consensus-based homogenization that Habermas assumes, contributes as fluctuation to the transformation of society*.

In fact, as Durkheim pointed out, people in modernity are so diversified that they share little but their humanity. That being so, the event probability of fluctuations cannot be reduced to zero. Moreover, for more stability and survivability, a system will do better dealing with the environment in the system’s own peculiar way, keeping a certain distance, than by adapting to the environment completely (see Luhmann 1990a: 556). A perfect, non-redundant adaptation at a certain point in time could make it hard to adapt to an environmental change in the future: *the death of the fittest*. This is the same as a medical system that experiences a neo-liberal reform, which, because of the lack of redundancy, collapses in an unexpected pandemic like the COVID-19 crisis (note that neo-liberalism is also a planned economy based on the belief that a rational state can perfectly calculate what is and will be unnecessary in advance).

So, for a specific way of differentiating from and responding to the environment, a system requires fluctuations as a clue to *deviation amplification* (via self-intermediate positive feedback) (see Luhmann 2000: 411–431). High homogeneity, as in a national society, might have been efficient for uniquely projected purposes such as industrialization (simple or linear modernization, in Ulrich Beck’s terms). However, in this current, so-called risk society with an increasingly uncertain future, high homogeneity cannot adequately deal with unknown events due to its lack of flexibility. Besides, the cost of suppressing fluctuations would grow. In this complicated, rapidly metamorphosing world society, planning cannot be conducted in a linear-causally secure manner.^6^ To be rational, one must rather amplify deviations from an existing channel that was taken for granted. In this light, today’s prevailing mindset that innovation will be achieved through “selection and concentration”—a mindset that can be named *national capitalism* after national socialism—is devoid of rationality. As is the case with a planned economy,^7^ this neo-liberal mindset is a “fatal conceit” that it is possible to reconstructively calculate all things by spatializing time. In reality, this vanity would ruin the seed of a deviant new combination or symbiotic relationship, thereby making it more improbable that an evolutionary leap occurs based on *élan vital*. Creative evolution arises from an indiscriminately streaming mixture in the present, that is, from a diverse and fluid complexity beyond a reconstructive calculation^8^: “assorted odds and ends” must, even if appearing to be useless at the time, be retained because evolution “feeds upon deviations from normal reproduction” (Luhmann 1990b: 180). Rationality makes sense only when taking this into account.

### Sociological Enlightenment and Evolution: Sociology in the Social Division of Labor

Society is indeed a continuously becoming reality, but its completely path-dependent, locked-in state like pure duration of the natural attitude cannot be called “change” in the usual sense. Discontinuity opposed to continuity, that is, a deviating leap, is perceived as a change and an evolution. However, the system must produce both continuity and discontinuity within itself, provided that it is self-referential (see Luhmann 1975b: 155–156). In other words, *“society changes” means that society changes itself* (*sich ändern*).^9^ Therefore, we should ask how unplanned structural change and, accordingly, diversification and complication become possible in society despite its operative closure (Luhmann 1993a: 240); in other words, how society can evolve by moving away from being submerged in the path-dependent pure duration and by replacing its own structure. As a matter of fact, returning to pure duration, as Bergson asserts, gives no guarantee that an individual’s variant fluctuation turns into society’s impulsive leap. Worse, the possibility even exists for the fluctuation to be oriented toward social devolution (retrogressive evolution). Rationality thus remains indispensable for social evolution, yet rational planning based on the reflection of the natural attitude would, as Schutz indicated about consciousness, result in a uniquely determined, self-bound state: As in the so-called first modernity, it could merely reduce a primordial diversity of the “pure duration of society” and make the otherwiseness of living experience and action more improbable. Unlike Durkheim, today, we cannot naively expect either that the central government enlightens society or helps it evolve. The will toward rational planning can expose individual freedom to the risk of unintended consequences, since it will by no means be realized as expected in this functionally differentiated, complex society.

Here, it is worth considering the role of sociology in the social division of labor. The main tasks of sociology can be summarized as the following. One is to inquire how a self-referential path-dependency (a spontaneous order) emerges from an accidental beginning (and how and what ideal or norm is chosen from there); this is formulated as the problem of social order. However, what is more important is the other: *to doubt self-evidence*. Its possible contribution to social evolution has been poorly understood. In actuality, it is inextricably linked to the problem of social order. That is, sociology reveals that the existing order deemed as self-evident and perhaps necessary is just a contingent order formed in a path-dependent manner, and thereby demonstrates the possibility of different living experiences and actions, as well as different ways of living. Thus, the discipline serves the evolution of society toward freedom by bracketing the “epoché of the natural attitude” in pure duration, not by planning or idealizing an order uniquely but by proving a fixed modern order to be contingent. A way of life (*Leben*; *vie*) that has neither been easy, allowed, nor even considered can thereby become an option.

Sociologically, there is no reason that women cannot become political leaders; that lesbian or gay couples cannot get married; and that Black people cannot live without worry of being shot by the police. Taken-for-granted, existing order of those kinds, which some people try to justify as necessary by scientific-looking grounds, is merely a path-dependently formed belief. In this sense, the task of the theory of self-referential social systems is epistemological as well. In fact, Luhmann’s theory inherits the phenomenological sociological view on the mundane world. As is known, Luhmann regarded self-referential social systems not as mere macro structural things (*choses*) confronting individuals but as autonomous observers who subjectively observe their environments from their own perspectives. What grounds a social system’s own ability of observing its environment is the *communicative intentionality*. As Tada (2010: 189, 2013a: chap. 2) clearly points out, *communication, of which a social system consists, is always a communication of something*, as phenomenology made a discovery about consciousness. There is a necessary intentional correlation between communication and external reality. Thus, while Durkheim (and Parsons, too) ontologically focuses on *what* the internal, ontological structure of society is, Luhmann poses an epistemological question about *how* each social system recognizes the external world. The inner side of a social system matters only for this task, because, as Schutz thought, how something external appears to a social system and why it appears so to the system now depends on the state of the inner time order of the system in question at that time (Tada [2018] 2019).

This *epistemological turn* by Luhmann in macro sociological theory can be thus regarded as an adaptation from Schutz’s phenomenological sociology of multiple social reality. Because of the difference of the inner time that each actor has formed path-dependently (or self-referentially), the same external world appears to each actor with a different subjective meaning. Based on this, Schutz stated that the observer of the social world is interested in

[…] neither the interpretation of *what* will be expressed, namely, the ideal objectivity of expression, nor its meaning that remains invariant no matter who has settled it. Instead, the observer of the social world seeks to interpret the phenomenon that it is just A [an actor] who accomplished this settlement now, here and so (*jetzt, hier und so*) (Schütz 1932: 32, emphasis in original).

In sum, the sociological observer observes how something appears to people who live with the epoché of the natural attitude in the mundane world. Further, because of communicative intentionality, this subjectivist approach is non-reductively applicable to social systems as well. For instance, Schutz’s term “epoché of the natural attitude” can directly correspond to Luhmann’s term “first-order observation” (Yamaguchi 2002: 189; Tada 2010: 194). According to Schutz, man in his natural attitude puts “the doubt that the world and its objects might be otherwise than it appears to him” (Schutz [1945] 1982: 229) in brackets. Likewise, insofar as being a first-order observer, a social system is in its pure duration and takes the coming and going of communication-correlated (social) phenomena only “as they give themselves,” without taking into account contingency (Tada [2018] 2019: 1001). This first-order observer’s attitude could be characterized as a naïve-realist’s one, in which the observed world appears as self-evident or even necessary. In contrast, Luhmann referred to the observer who observes the first-order observer as a *second-order observer*, who focuses on *how* something appears to a first-order observer subjectively in the now and so of her/his eigen-time, rather than *what* that something really objectively is.

In Luhmann’s opinion, the theory of self-referential social systems is primarily such a second-order observer (see, e.g., Luhmann 1995: chap. 2). The social systems theory brackets the social reality (cognitive order) that first-order observers firmly take for granted. In other words, it always accomplishes a phenomenological reduction. Luhmann remarks,

[In the social systems theory] it is not all about an interest in acknowledgment (*Anerkennnung*) and healing, nor about an interest in preserving the status quo, but first and foremost about an analytical interest: about breaking through the appearance of normality, about leaving aside experiences and habits, and in this (here non-transcendental theoretical) sense: about phenomenological reduction. (Luhmann 1984: 162)

As already discussed, disorder is impossible and a social order necessarily emerges in a self-reinforcing manner. In other words, some spontaneous order generates and becomes normal. It then often works as an “external and binding force” (Durkheim) or an “iron cage” (Weber) in which people’s freedom is limited; any new order, even if more complex, can increase new bindings as well as freedom again (see also Luhmann 1984: 435–436). Therefore, if sociology seriously hopes to receive an inheritance from the Enlightenment, it cannot exclusively see the constitution of social order. At the same time, the deviation amplification from the existing, taken-for-granted order becomes one of sociology’s contributions. Sociology searches for different possibilities that can *emancipate* the first-order observers from the ossified reality that appears invariant to and binds them.

This could be the re-definition of what Luhmann ([1967] 1970) referred to as “sociological enlightenment.” As a function that sociology bears in the social division of labor, we can here reformulate sociological enlightenment as the one that is always oriented toward the enlargement of otherwiseness, that is, the evolution of society. Contrary to the Spencerian worldview of the survival of the fittest, Luhmann originally conceived that other possibilities are not eradicated through selection but remain preserved in the meaning horizon. Even a variant option that was once denied as unfitting at a point in time is still retained and can be chosen anew and made probable (re-stabilized). Fluctuations springing from diversified individuals are in themselves accidental events that will vanish; for social evolution, they must be selected in society, path-dependently stabilized, and become probable and self-evident again (see Luhmann 1990a: 556–561, 1990b: 180, 1993a: 240, 277–278, 1997: chap. 3, 2000: 408–412, 2005: 188–190).^10^ Hence, sociology helps deviant fluctuations at the individual level gain social resonance through communication and acquire a socio-structural probability, insofar as they contribute to social evolution; thereby, society can use deviations as catalysts to reflexively find a way out of the path-dependent naïve belief and to change itself self-critically.

Note, however, that sociological enlightenment in no way aims to construct another ideal, moral order that some believe to be a single normatively correct order for social change. Such an elitist, enlightening desire, which homogenizes the social ideal by transcendently instructing from above or excluding supporters of an order “undesired” from its perspective as unenlightened people, is itself a social cleansing and the same as egoistic individualism that seeks to manipulate others.^11^ Indeed, it would even be normal for people to feel disaffected or anxious and look for an object of wrath with collective effervescence if the life order (or lifeworld) they had taken for granted became uncertain through upheavals as in these days. Nevertheless, if one simply asks people to sacrificially endure such a situation for the ideal from above, it is not much different from the worldview of an all-out fight. In fact, both the right and the left, respectively believing themselves to be true, seek as progress the transition toward a desirable order for their worldview. This is because both sides, regardless of ideology (dogmatized ideal), share humanism—the overconfidence in human ability that human beings can and should control society politically, that is, socio-technologically (see Moeller 2012: chap. 3).^12^

However, to not recognize the reality that perfect social planning is impossible is nothing other than a lack of intelligence or enlightenment. Human beings cannot change this complex, self-referential society, as intended: *Society has freedom from human beings*. It should be recalled that, in sociology, Durkheim had already delivered his criticism to the reasonable Enlightenment’s “simplistic rationalism” (Durkheim [1925] 2012: 229, 230) and “anthropocentric postulate” (Durkheim [1901] 1956: XXIII), regarding society as a reality *sui generis* with its own complexity (Durkheim [1925] 2012: 230). In this mundane world, everyone has only a selectively limited recognition with a blind spot. Neither “planning for freedom” (Karl Mannheim) nor the modernization of modernization by a sublime yet forced ideal can successfully alter society as intended. Rather, any enforcement could make individuals reflective and “awake” in the direction of their crowding into reactionary fluctuation or their seeking an irrational purpose through rational planning, as Schutz ([1957] 1976: 247) warned of a “vicious circle,” a negative spiral, between both camps. Such an unintended consequence can really happen if individuals are autonomous and diversified.

Therefore, sociological enlightenment first aims to *understand* (*verstehen*) multiple realities that appear in systems, whether those are “alternative facts” or the like—that is, to understand systems’ subjectivity (Tada 2013a: chap. 10) or, in Max Weber’s language, to understand “*what* the Divine (*das Göttliche*) is for the one order and for the other or: in the one and the other order” (Weber [1919] 1988: 604, emphasis in original). The second-order observation corresponds to this “understanding” that refers to observing how this complex world selectively appears to an observer. The pure duration of society should be inherently diversified, and sociology has no intention of homogenizing society by some ideal. *Sociologically, both “we” and “they” live in the same society together*: “There is an eigen-logic of the bad, which also belongs to the society and does not lie outside its boundaries” (Luhmann [1975] 2008: 213, see also Luhmann [1992b] 2008: 16). Hence, *sociology includes through understanding, as opposed to excluding*^13^: the understanding sociology (*verstehende Soziologie*) in a literal sense. However, as Weber ([1919] 1988: 503) also stated, understanding in this context means neither forgiveness nor approval; nor is it the cliché of pluralism (or multiculturalism) as the normatively correct answer.^14^ Rather, as discussed above, sociological enlightenment reveals the absence of one absolute, enlightening solution. In brief, it does not guide to the correct answer instructively but shows another perspective that is different from the one taken for granted as correct. Through understanding, it finds the limitations and blind spots of the first-order observers’ recognition and thereby shifts the way of seeing that they believe to be self-evident and even necessary.

In any case, understanding would help sociology learn multiple realities in society more deeply. In contrast, one-sided accusations from above which are done in the name of instruction, ignoring the *observer-relativity of reality* (environment), is not a scientific activity but a religious one; “ordinary people” have also noticed that such a privileged social position is no longer possible. Therefore, forcing an ideal without a listening attitude is only going to make matters worse. Rather, to dissolve the disaffection or anxiety widespread in society, understanding peoples’ social realities and thereby exploring the different perspectives that may be acceptable for them would be helpful. Sociological enlightenment is neither anti-rationalism, post-modernism, nor the abandonment of enlightenment; it is a dynamic, reflexive “Romani reason [*Vernunft der Zigeuner*]” (see Luhmann 1996: 45–46; Baecker 1999) that keeps on observing unobserved limitations and blind spots, including those of normative ideals such as “society should be so,” in this very age when both rightists and leftists are keen to change society into the (static and fixed) ideal order that they each desire (see also Luhmann 1997: 21–22, [1967] 1970, 1984: 654, 1990c: 260–261).

Sociological enlightenment starts from accepting the complexity of the world as it is, not from believing a type-simplified reality or ideal as the ontological reality; then, by “wandering around” this complex world, it goes on exchanging first-order observers’ beliefs in realities critically—of course, *self-critically* as well, because there are neither privileged nor transcendental positions in society (see Luhmann 1996: 45–46). In sum, sociological enlightenment finds rationality in never-ending skepticism toward path-dependent self-evidence. To be rational, any system must leave not only others’ realities, but also the reality that it itself holds, by reflexively moving out of the natural attitude. “The concept of rationality formulates only the most demanding perspective of the self-reflection of a system. It signifies no norm, no value, no ideal that surmounts real systems” (Luhmann 1984: 645, see also 638–646).

In facing social issues, there is no reason to believe that only one solution exists. Such determinism is the authoritarian-conformist dogma of lazy reason. Realistically, to think that other solutions, or alternatives to alternatives, are still in existence is much more reasonable and, besides, much more opened and *creative*. The thing to be stressed is that a limitation and blind spot of intelligence are rather a condition of the possibility of social evolution. The selectivity of a recognition perspective implies that a different way of viewing things is always possible. This was precisely what Luhmann meant by *functionalism*: other options remain and are not thrown away. To compare the existing choice with another and to exchange between them is always possible. This process will enlarge the otherwiseness of living experience and action—the room for freedom.

*Functionalist thinking will likely demand a new definition of the nature of human freedom*. The functionalist analysis does not fix the actor on a durably completed end of his action or on an accurately represented purpose. Nor does it attempt to explain the action causal-lawfully. It interprets the action under chosen, abstract, and therefore exchangeable points of view to make understandable that the act is [merely] a possibility among others. (Luhmann [1962] 1970: 27, emphasis added)

Thus, sociological enlightenment reconstructs not a normatively desirable order, but rather possibilities other than what is considered desirable: it makes a contribution to social evolution through *the reconstruction of contingency*, not that of necessity as in reasonable Enlightenment.^15^ In this sense, sociological enlightenment is the enlightenment of enlightenment, that is, *second-order enlightenment*, which continuously aims at enabling a new social order with enlarged, diversified possibilities of living experiences and actions by reconstructing contingency, not by negating it.

## Conclusion: Toward the Freedom of Society

In Luhmann’s opinion, contingency has already become a value peculiar to modernity—*the eigen-value of modernity* (Luhmann 1992a: 46–47, chap. 3).^16^ Indeed, value-polytheism is a reality of modern society and should be even upheld against value-monotheism: with the functional differentiation of society, a privileged regulatory authority has disappeared and, more importantly, *should no longer exist*. It may be said that modern society based on the value of contingency is normatively oriented toward differentiation rather than integration (Tada 2013a: 306). In fact, discussions about how to change society are also made possible only under the premise that the present society could be otherwise. That being so, contingency means neither imperfection nor less value but leads to creation (see Luhmann 1992a: 108). Contingency functions as the most comprehensive value that unifies diversity and enables further diversification (poly-contextualization); if sociology also follows some value (not anti-rationally but value-rationally), it would be contingency because of its *raison d’être* as a science of modern society.

As an unfixed Romani reason based on contingency, sociology makes modern society dynamic as a literally unfinished project for further freedom. Accordingly, sociology would be required to compare policies or behaviors in terms of whether they amplify otherwiseness; however, their belonging to liberalism, socialism, or conservativism does not matter in and of itself. Rather, no matter how lofty an ideal is, its dogmatization or fundamentalization leads to the devolution of society—even moral individualism, a modern belief in individual freedom, can oppress a religious minority in some situations today, being connected with the ultra-laicism (see also Tada 2020).^17^ In contrast, sociology that believes in contingency could state, as a pun on Rosa Luxemburg’s words, that *freedom is always the freedom in the differently thinking society* (*Freiheit ist immer die Freiheit in der andersdenkenden Gesellschaft*). Then, to widen the range of otherwiseness in living experience and action, it is more relevant to comparatively evaluate everything in terms of the contributions made to social evolution and, if needed, to revise or exchange the existing option. The expansion of freedom in this manner is the task of the sociological enlightenment of doubting self-evidence.

These implications of Luhmann’s social systems theory for social evolution seem to have been almost overlooked. This systems theory is by no means the stabilizing technology of dominance the Frankfurt school’s critical theorist accuses it of being. Conversely, it consistently aims to destabilize and make dynamic the existing order under the premise that the current state of society is exchangeable and reformable. Moreover, this orientation is always directed at helping society evolve rather than devolve, that is, to increase possible living experiences and actions in society.^18^ Thus, systems theory still works with enlightenment and rationality, but, as argued above, is no longer reasonable enlightenment from above. To this point, Durkheim’s statement about the role of sociology is relevant:

This one [social science], as with any science, studies that which is and that which was, looking for the laws, but disassociates itself from the future. […] Practical difficulties can be definitively settled only through the practice, through the experience of every day. It is not a meeting of sociologists but the societies themselves that find the solution. (Durkheim [1890] 1987: 225)

Contrary to the assumption of the reasonable enlightenment thinker, Durkheim thought that sociologists should not decide how society should change but *leave that to the free choice of society itself*. However, at the same time, he found it beneficial for sociologists to apply their reflections to actual matters, based on scientific results, and that the reflections reach people other than scientists, for instance, persons of action, as well (see Durkheim [1890] 1987: 225). This is one of the practical roles of sociology in the social division of labor.^19^ To begin with, social division of labor refers to the exchange of differences among people (and, accordingly, interdependence among them) on the premise of limited human ability; this was the reason that Durkheim aspired after moral individualism. The limit of human capability and, therefore, the individual selectivity of perspective are a prerequisite for the evolution of society, since they mean individuals’ individuality and its fundamental diversity. Therefore, as opposed to any kind of normative theory, sociological enlightenment amplifies deviations by bracketing the immobilized epoché of the natural attitude so that society evolves itself further toward freedom.

## Data Availability

Not Applicable.

## Footnotes

1 For instance, Durkheim certainly criticized Heinrich von Treitschke for his nationalist state theory in a war pamphlet (Durkheim 1915). He also had acknowledged in a philosophical conference that the “human fatherland (*patrie humaine*)” was being formed in Europe (Durkheim [1908] 1987: 294–295). However, Durkheim did not always think that existing national fatherlands should be overcome by the human one: As an ideal, the latter just covers the former. Durkheim thus flatly denied such a notion that national fatherlands will (or must) be completely sublated by some borderless collective like an enlarged, “internationalist” class-community of proletariats. “It is not difficult to make the worker understand that even her/his dearest aspirations always assume, as a necessary premise, a strongly organized fatherland; so much so that in trying to destroy fatherlands, s/he breaks with her/his own hands the only instrument that allows her/him to reach the goal s/he is striving for” (Durkheim [1908] 1987: 300). For Durkheim, the civilized state that embodies modern ideals remained a realistic backing to achieve humanity and the peace of mankind.

2 Terms such as path-dependency, self-reinforcement, or lock-in in this article are partially derived from Arthur (1994).

3 This distinguishes Schutz not only from Parsons but also from Edmund Husserl. A common feature of the latter two scholars is that they assumed the reality of the regulating ideal. See Tada (2013b).

4 To borrow Georg Simmel’s thought, society is not a *being* (society; *Gesellschaft*) but a *becoming* (sociation; *Vergesellschaftung*). See Tada (2013a: chap. 12).

5 Incidentally, quantitative and qualitative study make no fundamental difference with respect to focusing on this *significant bias* (Tada 2013a: chap. 7, fn. 3 and chap. 10, fn. 21 and 22).

6 With respect to planning, see Luhmann (1984: 635–638, 1990b: 179–181). And as for the metamorphosis of world society, see Beck (2016).

7 Hobsbawm ([1994] 1996: 411–413) and Graeber (2015) indicate the tendency for neo-liberal regimes to bloat bureaucratic works, such as documentary examinations to promote competition, and even to increase governmental expenditures due to nationalistic military expansion or poverty programs—poverty that was created by the government’s own policies. It also follows that bureaucrats’ powers grow.

8 As a suggestive work concerning this, see Florida (2012) and, theoretically, Arthur (2009).

9 Moeller (2006: 115) shows a similar view.

10 Luhmann explains a sequence of evolutionary mechanism, that is, variation, selection, and stabilization, but this paper does not dwell on it. See, for instance, Luhmann (1975b: 152–153, 1990a: chap. 8).

11 Jones ([2012] 2016) reports that hostility toward and demonization of the working class (in sum, hate for the working class) is widespread in the United Kingdom. Even liberal leftists are no exception: they sometimes bash the working class, based on the prejudice that working-class people are racists, ignoring both their ethnic diversity and class difficulties. That being so, it is not always unreasonable that such people challenged by a “class war from above” have antipathy toward the establishment, and recent populism in the West can also be considered *subpolitics* against a path-dependently re-feudalized society.

12 In addition, Moeller (2012: 28–31) positively evaluates as a “sociological insult” Luhmann’s radical functionalist viewpoint, which denies such socio-technological vanity.

13 As for this, see also Snyder’s ([2010] 2015: 399–401, 2015: 314–318) evocative remarks about genocides during World War II. Likewise, sociology today should also understand how, why, and what reality has appeared to those who made a “reactionary” choice in Western elections in recent years. Practically, populism can still be considered a democratic attempt to reset the asymmetrized political-economic configuration (cf. Haken [1981] 1990: 174–175) and even has some positive effects on democracy (Mudde and Kaltwasser 2017: chap. 5). Nevertheless, there is no reason to be too optimistic, as there is a relatively strong disposition toward tyranny of the majority and, accordingly, social devolution. A realistic countermeasure seems to understand and politically include citizens supporting a populist, that is, the demand side of populism and thereby to decrease the needs for it. A simplistic, idealistic attack on them would rather strengthen their “solidarity” (also with the supply-side populists). See Mudde and Kaltwasser (2017: chap. 6).

14 This does not mean a denial of that value itself; sociological enlightenment merely deals with value as a social reality. For instance, Nishikawa ([2005] 2006) indicates the possibility that multiculturalism is, as a state ideal, beneficial for the majority: by telling the future story of the coexistence of various cultures as one nation, it serves to contain minorities’ competing nation-building, hiding the majority’s inconvenient past. Moreover, pluralism evokes a sense of unfairness in some portion of the majority today, appearing as a form of elitism.

15 As for the reconstruction of contingency, see also Luhmann ([1973] 1975: 95 n. 4) and suggestively Luhmann (1984: 467–468). Regarding the above-mentioned relationship between sociological enlightenment and functionalism, see Luhmann ([1967] 1970: 75) as well.

16 Also, Luhmann (1990b: 134–135, [1993b] 2008: 240–245) suggests that the concept of eigen-value literally refers to an inviolate “value,” not a numerical value.

17 Symbolically, even Durkheim insisted that “[t]here could not be a question of granting to the majority the right to impose its [illuminative] ideas on the children of the minority” (Durkheim 1922: 62). In contrast, sociological enlightenment would mainly target the majority, not minorities: Since existing self-evidences in society are usually the ones for the majority, *it is rather the majority who must be first sociologically enlightened*. Yet, sociological enlightenment should not be an elitist “enlightenment from above.” What I discussed above as *understanding* is related to this point.

18 Sociological enlightenment is, of course, directed at the socio-economic divide as well. As Frank and Cook (1995) have clearly shown, a small difference in the beginning could self-reinforcingly produce a striking disparity later, as success breeds success: the *Matthew Effect*, in Robert K. Merton’s words. Seeing that even the initial condition of “competition” is unequal (asymmetrical), such path-dependency that cumulatively enlarges the rich-poor gap, restricting a group of people’s possibilities of living experiences and actions in their life-courses, can neither be overlooked nor justified in terms of social evolution.

19 Hence, a style difference like between “professional sociology” and “public sociology” should not always be viewed as confrontational. Sociology, as a *professional* second-order observer of modern society in the social division of labor, necessarily engages in a *public* mission of enlightenment for social evolution by reducing what is taken for granted in the existing systems and by providing alternatives that can be transformed to be probable. It is true that, *sociologically viewed*, sociology also has no privileged position in society and its rationality remains relative. However, such a self-reflection on its own recognition’s limitations prevents this discipline from clouding its eyes by self-dogmatization. I personally believe that the reflexive attitude derived from self-application is much more rational and professional. In this regard, sociologists would likely keep in mind the following statement by Luhmann: “Sociology must understand its relationship to society as a learning, not as a teaching” (Luhmann 1997: 22).
